# Dexmedetomidine-induced cardioprotection is mediated by inhibition of high mobility group box-1 and the cholinergic anti-inflammatory pathway in myocardial ischemia-reperfusion injury

**DOI:** 10.1371/journal.pone.0218726

**Published:** 2019-07-25

**Authors:** Juan Zhang, Fan Xia, Haifeng Zhao, Ke Peng, Huayue Liu, Xiaowen Meng, Chen Chen, Fuhai Ji

**Affiliations:** 1 Department of Anesthesiology, First Affiliated Hospital of Soochow University, Suzhou, China; 2 Department of Pathology, Suzhou Hospital Affiliated to Nanjing Medical University, Suzhou Science and Technology Town Hospital, Suzhou, China; The Second Affilated Hospital, Zhejiang University School of Medicine, CHINA

## Abstract

**Objectives:**

Dexmedetomidine (DEX) is a selective α2-adrenoceptor agonist that has anti-inflammatory and cardioprotective effects in myocardial ischemia/reperfusion (I/R) injury. The present study aimed to investigate the underlying mechanism by which DEX protects against myocardial I/R.

**Methods:**

Sprague Dawley rats were subjected to either sham operation or myocardial I/R, which was induced by ligating the left anterior descending coronary artery for 30 min followed by reperfusion for 120 min. Rats were treated with either DEX or saline prior to surgery. We measured heart infarct size, serum cardiac Troponin I (cTnI), cardiac High mobility group box-1 (HMGB1) expression, myocardial apoptosis and cytokine production of interleukin-6 (IL-6) and tumor necrosis factor-α (TNF-α). Besides, we evaluated the heart function at 4 weeks post-reperfusion by echocardiography. Unilateral vagotomy or inhibition of the α7 nicotinic acetylcholine receptor (α7nAChR) with methyllycaconitine (MLA) was applied to investigate whether DEX-induced cardioprotection is mediated via the cholinergic anti-inflammatory pathway. Cardiac-selective overexpression of HMGB1 was administered to further confirm if HMGB1 is a key anti-inflammatory target during DEX-induced cardioprotection.

**Results:**

DEX pretreatment significantly attenuated I/R-induced cardiac damage, as evidenced by decreases in short-term injury indicators including myocardial infarct size, cTnI release, myocardial apoptosis, cardiac HMGB1 expression, IL-6 and TNF-α production, as well as improvement on long-term cardiac function at 4 weeks post-reperfusion. These effects were partially reversed by either unilateral vagotomy or methyllycaconitine treatment. Besides, cardiac HMGB1-overexpression nearly abolished DEX-induced cardioprotection.

**Conclusions:**

DEX pretreatment protects against myocardial I/R by inhibiting cardiac HMGB1 production and activating the cholinergic anti-inflammatory pathway.

## Introduction

Ischemic heart diseases remain the most frequently reported threat to human health despite advances in pharmacological and interventional therapies in the past decades [1]. Restoring blood supply to the ischemic heart is the most effective clinical strategy for preserving viable myocardial tissue. However, secondary injuries after recanalization, referred to as myocardial ischemia-reperfusion (I/R) injury, can aggravate structural and functional myocardial damage. During myocardial I/R, the sympathovagal balance is impaired, characterized by sympathetic predominance and vagal depression [2]. Accumulating evidences indicate that directly or indirectly upregulating vagal tone reduces incidence of severe arrhythmias, alleviates inflammation and histological lesions [3–5].

Dexmedetomidine (DEX) is a highly selective α2-adrenoceptor agonist with sedative, analgesic, and anxiolytic effects which is widely used in clinical anesthesia and intensive care units [6, 7]. Our previous work demonstrated that DEX alleviates myocardial injury and improves cardiac outcomes during cardiac surgery [8]. Subsequent studies indicated that these beneficial effects of DEX may be associated with its anti-inflammatory properties [9, 10]. Using a lipopolysaccharide (LPS)-induced endotoxemia model, Xiang et al [11] found that DEX regulates systemic cytokine levels by activating the cholinergic pathway. Similarly, we observed that DEX attenuated neuroinflammation in a rat tibia fracture model [12]. Thus, we speculated that the anti-inflammatory effect of DEX is associated with its regulation towards the autonomic nervous system and restoring the sympathovagal imbalance.

High mobility group box-1 (HMGB1) is generally known as a non-histone DNA binding protein that is involved in DNA stabilization and gene transcription. However, following ischemia, HMGB1 can be passively released by necrotic cardiomyocytes or be actively generated by immune cells such as macrophages, monocytes, and dendritic cells [13, 14]. HMGB1 release in the initial phase of myocardial ischemia is an early mediator of inflammation during myocardial I/R [13]. Interestingly, our previous research indicates that triggering the vagal anti-inflammatory pathway by enhancing vagal tone can significantly reduce I/R-induced release of HMGB1 and ameliorate inflammation [5].

Although the potential cardioprotective effect of DEX has been reported both in clinical observations and animal experiments, the underlying mechanisms remain undefined. Our recent study demonstrated that DEX pretreatment reduced HMGB1 release and decreased the downstream Toll like receptor 4—Myeloid differentiation primary response 88—Nuclear factor-κB (TLR4-MyD88-NF-κB) signaling cascade in a rat myocardial I/R model [10]. However, the upstream pathway via which DEX modulates HMGB1 release and activates the subsequent inflammatory cascades in myocardial I/R injury requires further exploration. Hence, in this study, we investigated if DEX pretreatment attenuates myocardial I/R injury by modulating cardiac HMGB1 release via activating the cholinergic anti-inflammatory pathway.

## Materials and methods

### Animals

All procedures involving experimental animals were approved by the Ethics Committees of the Animal Center, Soochow University, Suzhou, China (Affidavit of Approval of Animal Use Protocol No. 2016101801). All animal experiments were performed in accordance with the Guide for the Care and Use of Laboratory Animals of the National Institutes of Health. Adult male Sprague Dawley rats weighing 230–250 g (purchased from Xinuosai Biological Technology Ltd., Suzhou, China) were maintained under a 12-hour light/dark cycle at 25 ± 1°C with unrestricted access to standard food and water. All surgeries were performed under sodium pentobarbital anesthesia (50mg/kg, i.p.), and all efforts were made to minimize animal suffering. At the end of this study, carbon dioxide euthanasia was performed on all animals.

### Experiment design

**Fig 1:**
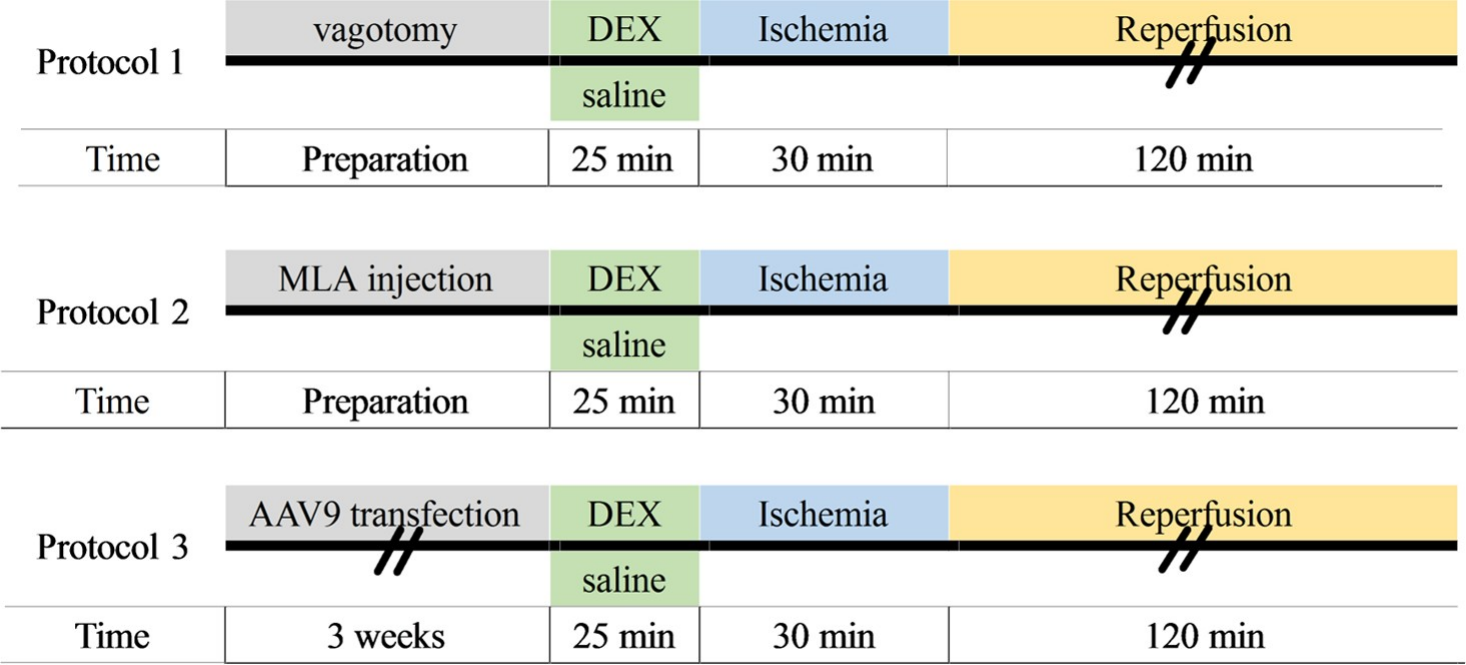
Schematic diagram of experimental design. Rat hearts were subjected to 30 min anterior descending coronary artery occlusion and 120 min of reperfusion. In protocol 1, unilateral vagotomy or sham operation was induced before DEX or saline pretreatment followed by myocardial I/R to determine the involvement of the vagal nerve. In protocol 2, MLA or saline was administered before DEX pretreatment to detect the involvement of the α7nAChR. In protocol 3, rats received an intrapericardial injection of AAV9-HMGB1 or AAV9-Blank and were subsequently subjected to either DEX or I/R treatment after 3 weeks. MLA: methyllycaconitine; AAV9: adeno-associated virus serotype 9; HMGB1: High mobility group box-1.

The experimental design is schematically represented in Fig 1.

#### Experimental protocol 1

To determine if the vagal nerve is involved in DEX-induced cardioprotection, rats were randomly divided into five groups (n = 10 per group): 1) Sham group (S); 2) Myocardial I/R group (I/R); 3) Vagotomy + I/R group (V+I/R); 4) DEX pretreatment + I/R group (D+I/R); and 5) Vagotomy + DEX + I/R group (V+D+I/R). For rats in the V+I/R and V+D+I/R groups, the right cervical vagal nerve was isolated and snipped. The rats were then treated with DEX (1 μg/kg/h x 10 min + 0.7 μg/kg/h x 15 min, intravenously) or with the same volume of saline before undergoing myocardial I/R. For rats in the S group, the right cervical vagal nerve was isolated but not snipped.

#### Experimental protocol 2

To determine the role of α7 nAChR in DEX-induced cardioprotection, rats were randomly divided into five groups (n = 10 per group):1) Sham group (S); 2) Myocardial I/R group (I/R); 3) DEX pretreatment + I/R group (D+I/R); 4) Methyllycaconitine + DEX + I/R group (M+D+I/R); and 5) Methyllycaconitine + I/R group (M+I/R). In the M+D+I/R and M+I/R groups, a selective α7nAChR antagonist, methyllycaconitine (MLA; 10 mg/kg), was intraperitoneally injected before DEX treatment or myocardial I/R.

#### Experimental protocol 3

To determine if HMGB1 is the target of the DEX-mediated anti-inflammatory response during myocardial I/R, rats were randomly divided into four groups (n = 10 per group): 1) Myocardial I/R group (I/R); 2) DEX pretreatment + I/R group (D+I/R); 3) HMGB1-overexpression + DEX + I/R group (H+D+I/R); and 4) Blank-control + DEX + I/R group (B+D+I/R). HMGB1 overexpression was achieved by intrapericardially injecting 500 U hyaluronidase, 0.7 mg collagenase, and 4 x 10^11^ Adeno-Associated Virus 9 (AAV9)-HMGB1 mixture; the Blank control received equivalent hyaluronidase, collagenase, and blank AAV9.

### Myocardial I/R model

Rats were subjected to myocardial I/R as previously described [15]. Briefly, rats were intraperitoneally anesthetized with 1% pentobarbital sodium (50 mg/kg, purchased from Sigma, St. Louis, USA), intubated, and ventilated with a rodent ventilator (ALC-V8, Shanghai, China). Efficacy of anesthesia was determined using a tail pinch test prior to the surgical procedure. Catheters were inserted into the carotid artery to monitor vital signs using the MedLab software (Nanjing, China). Lateral thoracotomy was performed at the fourth and fifth intercostal spaces, and the left anterior descending artery (LAD) was ligated using a 6.0 silk suture 2 mm below the boundary of the left auricle. The LAD ligation was maintained for 30 min (ischemia) and then released to achieve reperfusion for 120 min. The ischemia criteria included pale colored myocardium and electrocardiogram changes. The S group underwent the same operation but the LAD was not occluded. The incision in the chest wall was sutured with 5.0 non-absorbable silk sutures. Body temperature was maintained at 37°C by placing the rats on a heating pad (Physitemp Instruments, USA) throughout the study.

### Cardiac-selective HMGB1 overexpression

We introduced exogenous HMGB1 into myocardium using adeno-associated virus serotype 9 (AAV9)-mediated delivery system for cardiac-selective HMGB1 overexpression. In brief, the HMGB1 cDNA sequence conjugated to green fluorescent protein (GFP) was cloned into a pAAV ITR-containing plasmid driven by the CMV promoter (abm, Richmond, Canada; the vector map is shown in S1 Fig). Then a triple-transfection method with CaPO4 precipitation in HEK293 cells allowed for pAAV-ITRs to be packaged into AVV9. After the vectors were purified and concentrated, we obtained AVV9-HMGB1 and its blank control AVV9-Blank particles for subsequent animal protocol. First, a small oblique thoracotomy was performed lateral to the midsternal line in the fourth intercostal space to expose the heart. Second, a total of 4 × 10^11^ particles of AAV9-HMGB1, 0.7 mg collagenase, and 500 U hyaluronidase in a final volume of 700 μl were intrapericardially injected with a 30-gauge needle as previously described [16, 17]. Blank control rats received an equivalent volume of either sterile saline or AAV9-Blank. After injection, the exposed heart was monitored for 3 to 5 min to ensure a return to normal sinus rhythm. Negative intrapleural pressure was re-established by evacuating air from the chest using small tube syringe aspiration before chest closure. Three weeks after injection, the animals were subjected to the I/R operation.

### Infarct size determination

Infarct size was evaluated using Evans blue and 2, 3, 5-triphenyltetrazolium chloride staining (TTC) (Sigma-Aldrich, St. Louis, USA) as previously described [18]. Briefly, rats were anesthetized after 120 min of reperfusion with the LAD re-occluded. We then injected 1 ml of 2% Evans blue via the aortic arch. The heart was harvested and sliced transversely into sequential 2 mm-thick sections, incubated in 1% TTC at 37°C for 30 min, and fixed in 10% formalin overnight. The left ventricular (LV) area, area at risk (AAR), and infarct area (IA) were determined using ImageJ (National Institutes of Health, USA) and adjusted for weight. Images were captured using a microscope (DFC500, Leica, Germany) equipped with a digital camera (C-DSD230, Nikon, Japan). AAR/LV and IA/AAR were calculated using the following formulas:

$$\frac{\text{IA}}{\text{AAR}}=\frac{W_{1}\times I_{1}+W_{2}\times I_{2}+W_{3}\times I_{3}+W_{4}\times I_{4}+W_{5}\times I_{5}}{W_{1}\times A_{1}+W_{2}\times A_{2}+W_{3}\times A_{3}+W_{4}\times A_{4}+W_{5}\times A_{5}}$$

$$\frac{\text{AAR}}{\text{LV}}=\frac{W_{1}\times A_{1}+W_{2}\times A_{2}+W_{3}\times A_{3}+W_{4}\times A_{4}+W_{5}\times A_{5}}{W_{T}}$$

where *W*_*n*_ represents the weight of each heart section; *W*_*T*_, the weight of the entire left ventricle; *In*, percentage of infarct area (IA) (white areas) on each section; and *A*_*n*_, the percentage of area at risk (AAR) (white and red areas) on each section. IA/AAR indicates the severity of myocardial injury while a consistent AAR/LV means the stability of the myocardial I/R model.

### Echocardiography

Echocardiography was performed using the Vevo 770 high-resolution echocardiographic system (Visual Sonics Inc., Toronto, Canada) at 4 weeks after myocardial ischemia and reperfusion as previously described [19]. Animals were anesthetized with intraperitoneally injection of 1% pentobarbital sodium and placed in supine position. M-mode echocardiology was performed in the parasternal short and long-axis view. LV systolic function was determined by ejection fraction (EF): EF (%) = [(EDV−ESV)/EDV]×100%; EDV (end diastolic volume); ESV (end systolic volume). All measurements were based on the average of three consecutive cardiac cycles.

### Apoptosis assay

Myocardial apoptosis was examined by the TdTmediated dUTP nick end labelling (TUNEL) assay (Beyotime Biotechnology Ltd., Shanghai, China) as described previously [20]. In order to test the apoptotic rate in infarct zone, five visual fields were chosen randomly from each section, positive brown cells and total cells were counted by Image J (National Institutes of Health, USA). Apoptosis index (positive cell/ total cells×100%) was used as the indicator of apoptosis and was calculated for statistical analysis.

### Western blot analysis

After 120 min of reperfusion, hearts were rapidly harvested, placed in liquid nitrogen, and stored at -80°C until analysis. Each sample was homogenized for 3 min with RIPA containing PMSF (Beyotime Biotechnology Ltd., Shanghai, China) using a homogenizer (T10 basic ULTRA-TURRAX, Germany), and the supernatant was centrifuged at 12,000 rpm for 30 min at 4°C. Briefly, 20 μg of frozen samples were separated by 12% SDS-PAGE and transferred to a PVDF (Millipore Corp., Bedford, MA) membrane. The membranes were blocked with 5% nonfat milk in a universal antibody dilutent (New Cell& Molecular Biotech Co. Ltd., Suzhou, China) for 2 h at room temperature and incubated with primary antibodies against HMGB1 (1:1,000; Abcam, Massachusetts, USA) or glyceraldehyde phosphate dehydrogenase (GAPDH) (1:1,000; abm, Richmond, Canada) at 4°C overnight. The membranes were incubated for 2 h with HRP-conjugated secondary antibodies (1:1,000, Beyotime Biotechnology Ltd., Shanghai, China). Specific protein bands were visualized using an enhanced chemiluminescence (ECL) kit and then processed using Image J for quantification.

### Quantitative real-time PCR (qRT-PCR) analysis

Total RNA from rat hearts was extracted using the Trizol Reagent (Thermo Fisher, USA) according to the manufacturer’s instructions. Target cardiac gene expression was reverse transcribed into cDNA using EvaGreen (abm, Richmond, Canada). Primers were designed as follows: Interleukin-6 (IL-6): 5′—CCAGTTGCCTTCTTGGGACT—3′ and 5′—GGTCTGTTGTGGGTGGTATCC—3′; tumor necrosis factor-α (TNF-α): 5′—ATGGGCTCCCTCTCATCAGT -3′ and 5′—GCTTGGTGGTTTGCTACGAC -3′; HMGB1: 5′—CGCGGAGGAAAATCAACTAA—3′ and 5′—GGGTGCTTCTTCTTGTGCTC—3′; GAPDH as control: 5’–GGTTGTCTCCTGCGACTTC—3’ and 5’- CCTGTTGCTGTAGCCGTATTCAT—3’. Data were quantified automatically using the Light Cycler 480 (Roche, Switzerland) and the relative expression was determined using the 2^-ΔΔCt^ method.

### Immunohistochemistry analysis

Hearts fixed in 4% paraformaldehyde were embedded in paraffin and then cut into 5 μm sections. Sections were incubated with an anti-HMGB1 rat polyclonal primary antibody (1:200 dilution, 60 min at room temperature; Abcam, USA) and a secondary antibody (1:100 dilution, 60 min at room temperature; Zhongshan Biotechnology, China). The sections were incubated in HRP-streptavidin (1:100; Zhongshan Biotechnology, China) for 30 min at 37°C, and the color reaction was visualized with diaminobenzidine (DAB; ZSGB-BIO, Beijing, China). For each section, five fields were randomly selected from the infarcted area of the left ventricle and photographed with a digital camera (AF6000, Leica, Germany). The mean density of HMGB1 expression in the myocardium was analyzed using Image J.

### Enzyme-linked immunosorbent assay (ELISA)

All serum specimens were collected and frozen at -80°C before assays were performed. Serum levels of IL-6, TNF-α, and HMGB1 were assayed using ELISA kits (Multi Sciences Biotech Co., Ltd. Hangzhou, China) specific for rat. Serum levels of cTnI were assayed using an ELISA kit (Life diagnostics, Lincoln, USA). All analyses were carried out according to the manufacturer’s instructions.

### Statistical analysis

Data are expressed as mean ± standard error of the mean (SEM) and analyzed by one-way ANOVA, followed by Bonferroni correction for multiple comparisons. The threshold for significance was set at p < 0.05. All of the data were analyzed and graphed using GraphPad Prism 7 software (GraphPad Prism Institute Inc, La Jolla, USA).

## Results

### DEX-induced cardioprotection depends on vagal nerve integrity

**Fig 2:**
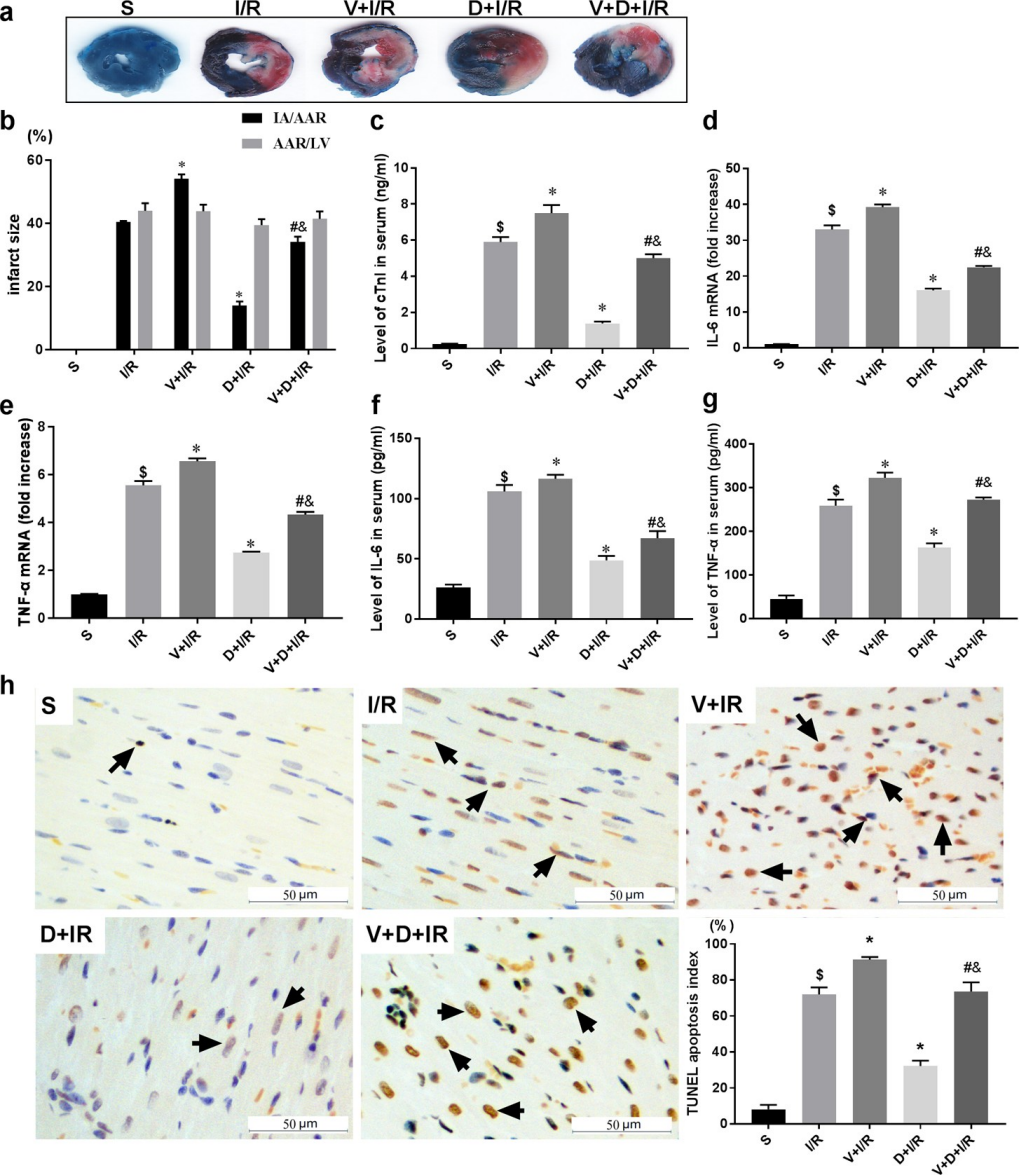
The vagal nerve mediates DEX-induced short-term cardioprotection against I/R. Unilateral vagotomy or sham operation was induced before DEX or saline pretreatment followed by myocardial I/R. The extent of myocardial injury was evaluated by infarct size using Evans blue/TTC staining (a-b), serum levels of cTnI (c), and myocardial expression of IL-6 and TNF-α (d-e). Serum levels of IL-6 and TNF-α were also measured by ELISA (f-g). Myocardial apoptosis in infarct zone was evaluated by TUNEL staining (h). Representative pictures for each group as well as the corresponding quantitative analysis using the TUNEL apoptosis index (TUNEL-positive cell numbers /total cell numbers ×100%) were shown; arrows indicate positive cells, Scale bar = 50μm. Data shown are mean ± SEM; n = 10 per group; $ p<0.05 vs. S; *p < 0.05 vs. I/R; #p < 0.05 vs. D+I/R; &p < 0.05 vs. V+I/R. All specimen were obtained at the timepoint of myocardial I/R for 2hrs. S: sham operation; I/R: myocardial ischemia-reperfusion; D: dexmedetomidine; V: vagotomy;

**Fig 3:**
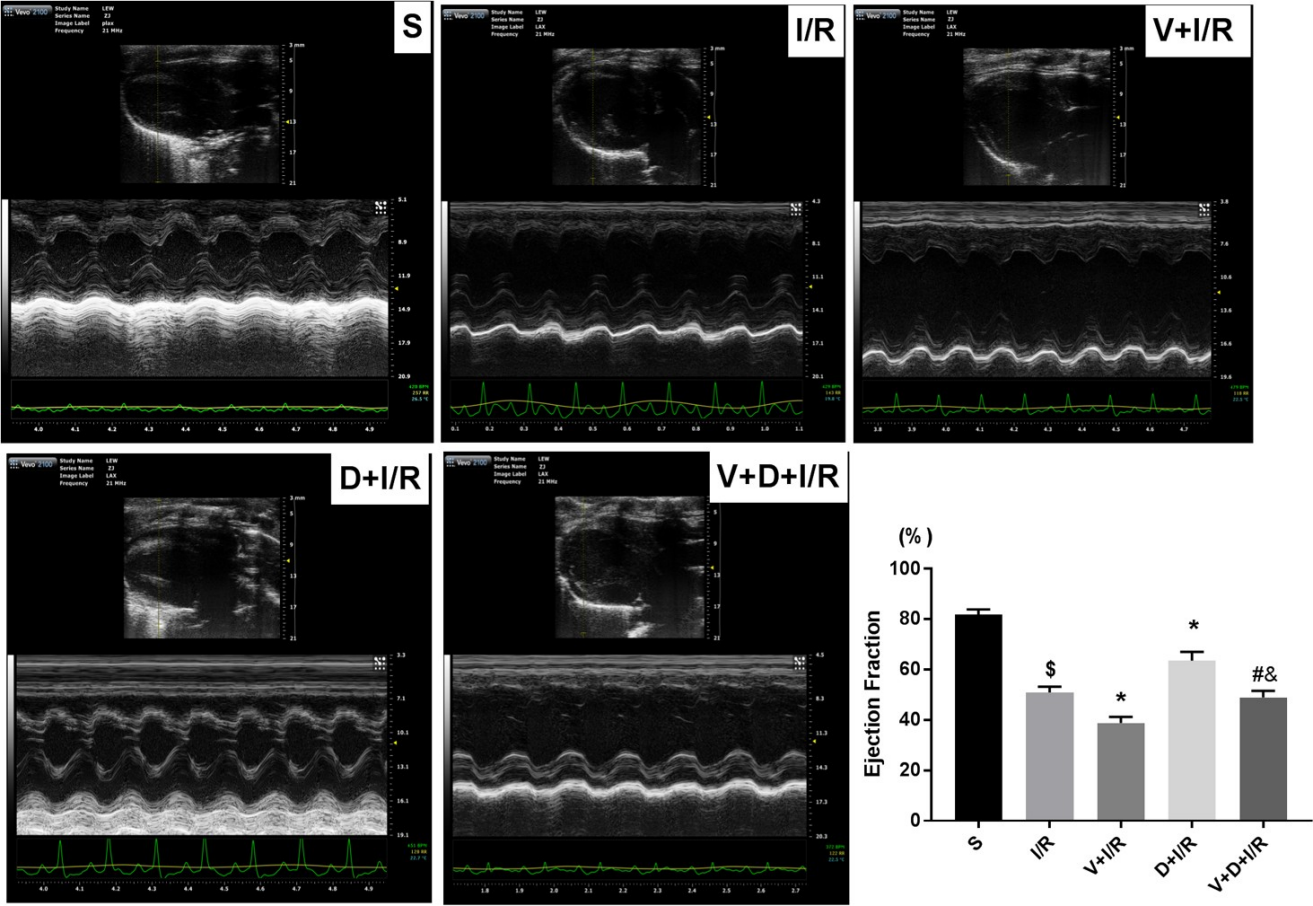
The vagal nerve mediates DEX-induced long-term cardioprotection against I/R. To investigate whether DEX exerts long-term cardioprotective effects and the involvement of the vagal nerve, echocardiography was performed to evaluate the LV function at 4 weeks post-I/R before animals were sacrificed. M-mode images at long-axis view were shown. Ejection fraction (EF) were calculated and compared among different groups: EF (%) = [(EDV−ESV)/EDV]×100%; EDV (end diastolic volume); ESV (end systolic volume). All measurements were based on the average of three consecutive cardiac cycles. Data shown are mean ± SEM; n = 6 per group; $ p<0.05 vs. S; *p < 0.05 vs. I/R; #p < 0.05 vs. D+I/R; &p < 0.05 vs. V+I/R. S: sham operation; I/R: myocardial ischemia-reperfusion; D: dexmedetomidine; V: vagotomy.;

**Fig 4:**
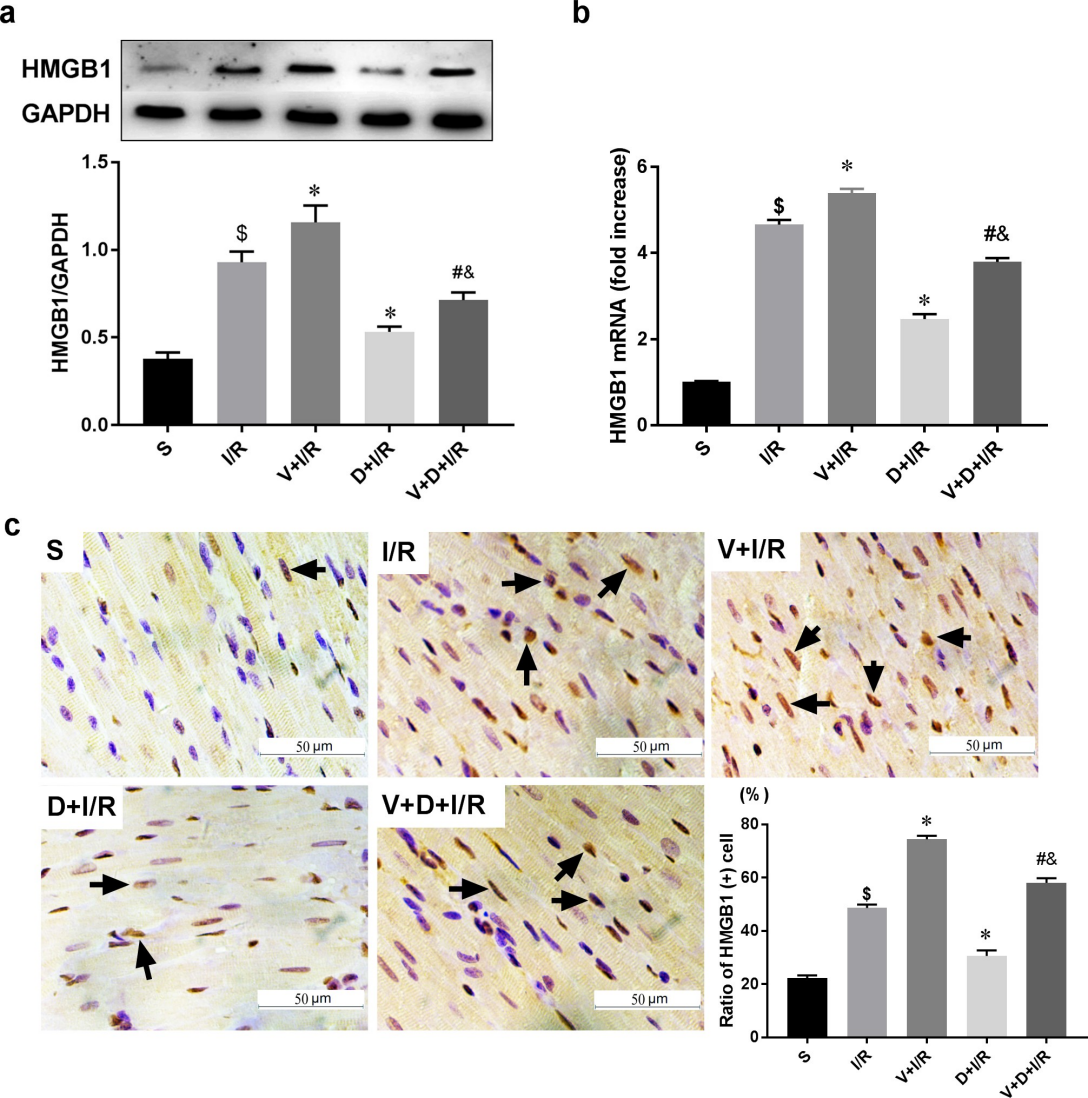
The vagal nerve modulates DEX-induced downregulation of cardiac HMGB1 during I/R. Unilateral vagotomy or sham operation was induced before DEX or saline pretreatment followed by myocardial I/R. HMGB1 protein levels in the left ventricles were measured using Western blot (a) and HMGB1 mRNA levels were assessed by qRT-PCR (b). Representative images of immunohistochemical staining for HMGB1 and quantitative results (c). Arrows indicate HMGB1-positive cells. Scale bar = 50μm. All specimen were obtained at the timepoint of myocardial I/R for 2hrs. Data shown are mean ± SEM; n = 6 per group; $ p<0.05 vs. S; *p < 0.05 vs. I/R; #p < 0.05 vs. D+I/R; &p < 0.05 vs. V+I/R. S: sham operation; I/R: myocardial ischemia-reperfusion; D: dexmedetomidine; V: vagotomy;

To determine if the cholinergic pathway is involved in DEX-induced cardioprotection, we treated rats with DEX prior to myocardial I/R with or without right unilateral vagotomy. We compared myocardial infarct size, serum levels of cTnI, and expression of HMGB1, TNF-α, and IL-6 in the myocardium and serum in all groups after 120 min of reperfusion. Consistent with our previous study, DEX pretreatment conferred significant cardioprotection against myocardial I/R as evidence by reduced infarct size (13.86 ± 2.45% in D+I/R *vs*. 41.88 ± 3.53% in I/R, p < 0.05; Fig 2A and 2B), decreased cTnI levels (D+I/R *vs*. I/R, p < 0.05; Fig 2C), reduced systematic and local IL-6 and TNF-α release (D+I/R *vs*. I/R, all p < 0.05; Fig 2D–2G) and attenuated myocardial apoptosis (D+I/R *vs*. I/R, p < 0.05; Fig 2H). Besides, we investigated the long-term effect of DEX by measuring the LV function after 4 weeks of reperfusion. Consistently, DEX pretreatment partially restored the I/R-induced LV dysfunction reflected by a significant increase of ejection fraction (EF, D+IR *vs*. I/R, p<0.05; Fig 3). DEX pretreatment also inhibited both cardiac HMGB1 mRNA levels and protein expression during myocardial I/R (data of Western blot, q-RT-PCR and immunohistochemical staining are shown in Fig 4A–4C). Vagotomy partially abolished the above effects of DEX: infarct size, cTnI serum levels, cytokine leakage, myocardial apoptosis and cardiac HMGB1 expression were significantly increased while the LV cardiac function at 4 weeks of reperfusion was worsen (V+D+I/R *vs*. D+I/R, p < 0.05; Figs 2–4). Vagotomy alone increased cardiac damage, including infarct size and cTnI levels, as well as HMGB1 expression (V+I/R *vs*. I/R, p < 0.05, Figs 2–4). These results suggest that the vagal nerve is involved in the process while DEX inhibits cardiac HMGB1 release and confers cardioprotection during myocardial I/R. Thus, we speculated that DEX-mediated vagal anti-inflammation could be blocked by inhibiting the α7-subunit of the nicotinic acetylcholine receptor (α7nAchR), which acts as a pivotal member in the cholinergic anti-inflammatory pathway.

### α7nAchR antagonism abolishes DEX-induced cardioprotection and inhibition of HMGB1 release in I/R

**Fig 5:**
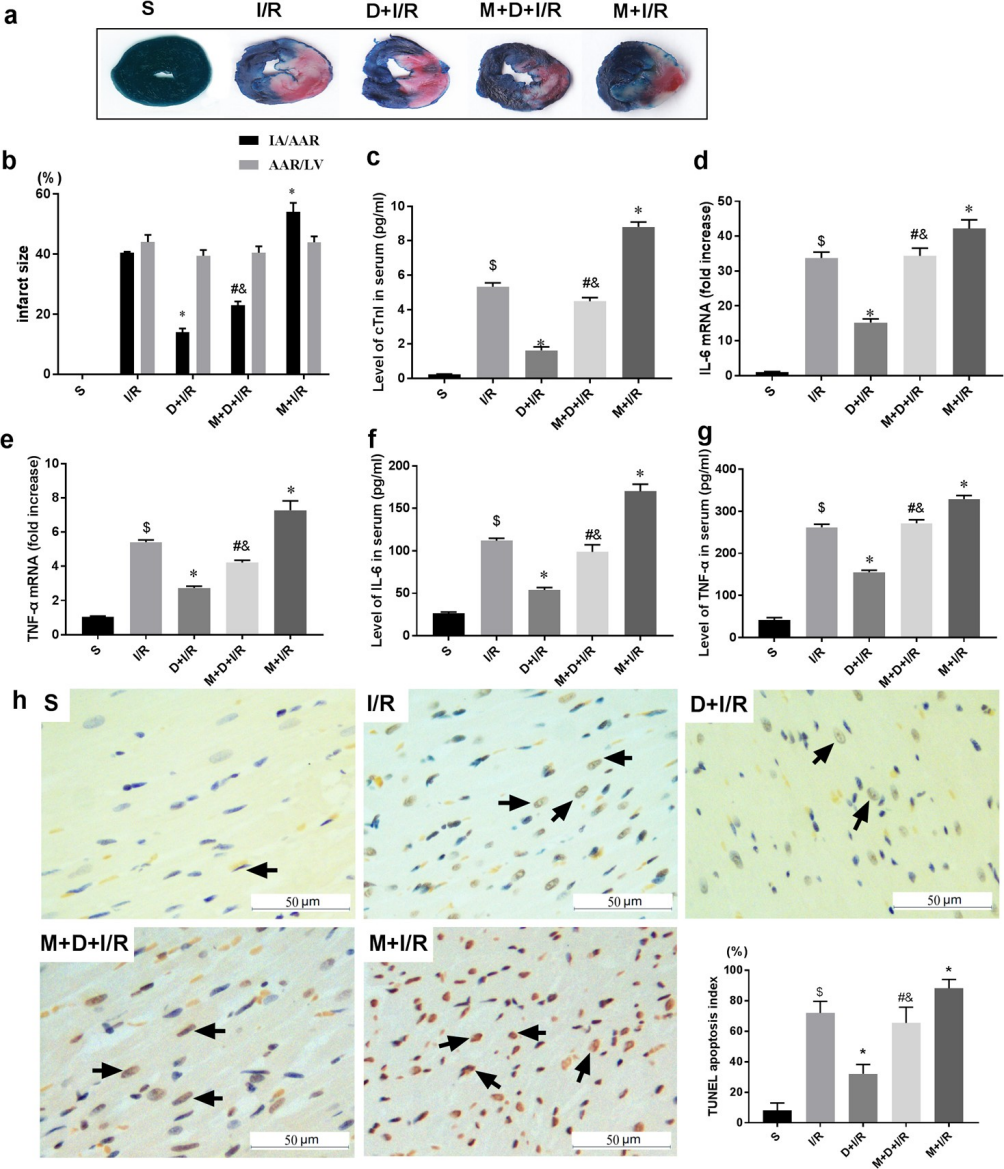
Blockade of the α7nAChR abolishes DEX-induced short-term cardioprotection against I/R. Rats were subjected to intraperitoneal injection of saline or methyllycaconitine (MLA, α7nAChR antagonist) before DEX treatment. (a-b) Infarct sizes determined by Evans blue/TTC staining and representative images of each group are shown. (c) Serum cTnI levels; mRNA levels (d-e) and serum levels (f-g) of inflammatory cytokines including IL-6 and TNF-α were also detected. Myocardial apoptosis in infarct zone was evaluated by TUNEL staining (h) (arrows indicate positive cells, Scale bar = 50μm.); Data shown are mean ± SEM; n = 10 per group; $ p<0.05 vs. S; *p < 0.05 vs. I/R; #p < 0.05 vs. D+I/R; &p < 0.05 vs. M+I/R. All specimen were obtained at the timepoint of myocardial I/R for 2hrs. S: sham operation; I/R: myocardial ischemia-reperfusion; D: dexmedetomidine; M: methyllycaconitine.

**Fig 6:**
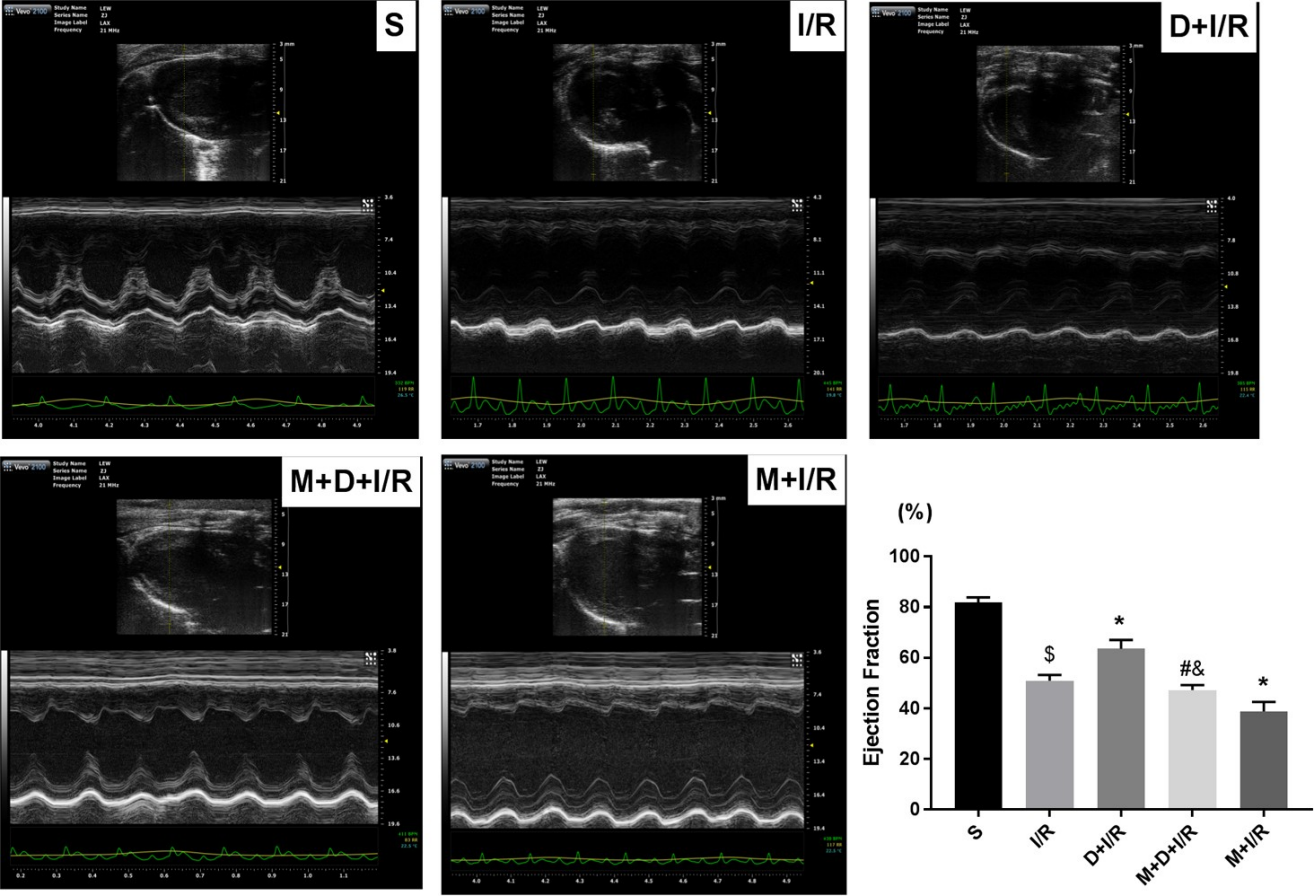
Blockade of the α7nAChR abolishes DEX-induced long-term cardioprotection against I/R. To investigate whether the α7nAChR was involved in the long-term cardioprotective effects of DEX, we administrate MLA, the selective antagonist of α7nAChR, prior to DEX treatment followed with I/R operation. After 4 weeks, the echocardiography was performed to evaluate the LV function before the animals were sacrificed. M-mode images at long-axis view were shown. Ejection fraction (EF) were calculated and compared among different groups: EF (%) = [(EDV−ESV)/EDV]×100%; EDV (end diastolic volume); ESV (end systolic volume). All measurements were based on the average of three consecutive cardiac cycles. Data shown are mean ± SEM; n = 6 per group; $ p<0.05 vs. S; *p < 0.05 vs. I/R; #p < 0.05 vs. D+I/R; &p < 0.05 vs. M+I/R. S: sham operation; I/R: myocardial ischemia-reperfusion; D: dexmedetomidine; M: methyllycaconitine.

**Fig 7:**
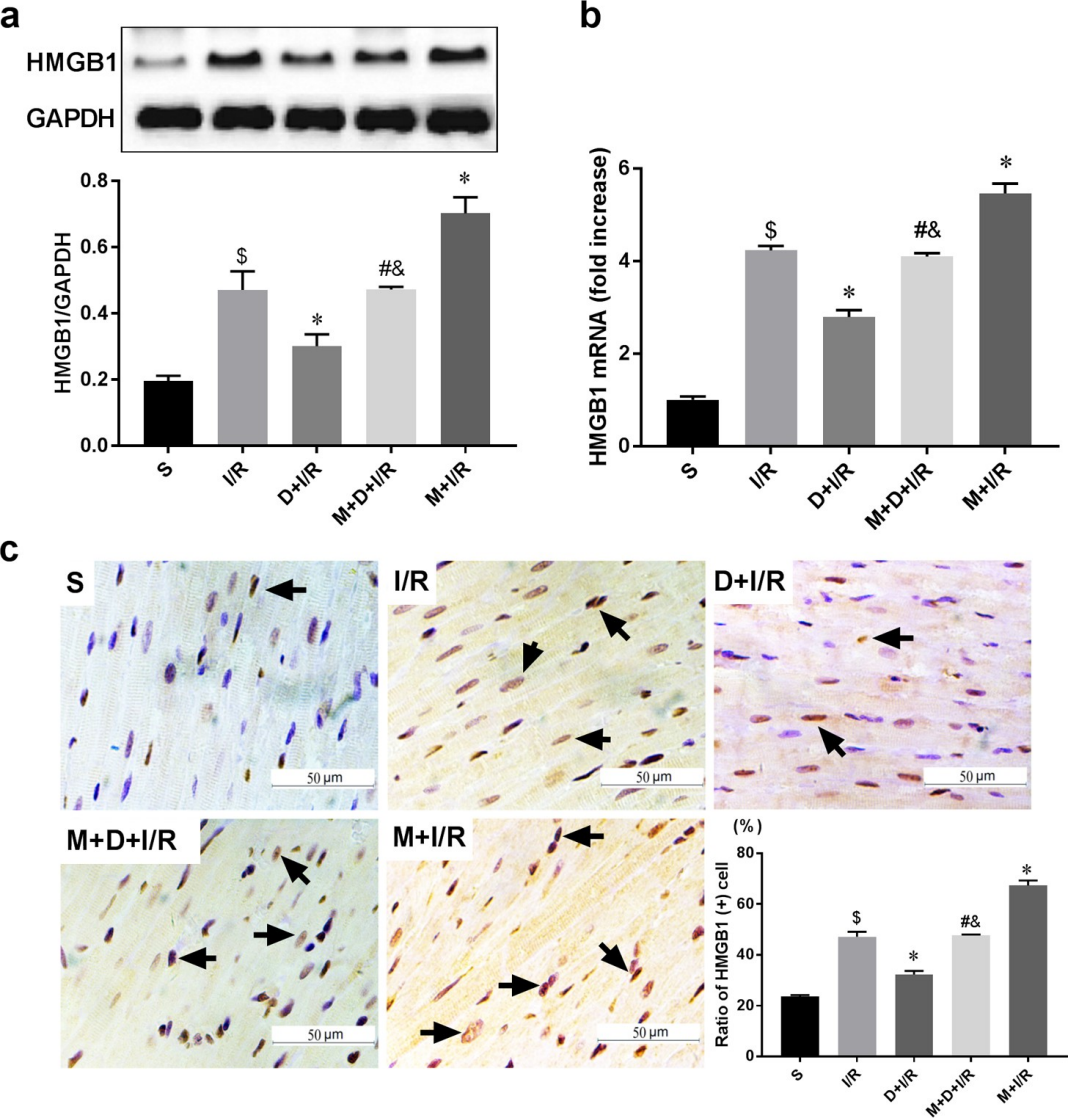
Blockade of the α7nAChR reverses DEX-induced downregulation of HMGB1 during I/R. To explore the involvement of α7nAChR, methyllycaconitine (MLA, α7nAChR antagonist) was administered. HMGB1 protein levels in the left ventricles were measured using Western blot (a) and HMGB1 mRNA levels were assessed by q-RT-PCR (b). Representative images of immunohistochemical staining for HMGB1 and quantitative results (c). Arrows indicate HMGB1-positive cells. Scale bar = 50 μm; n = 6 per group; $ p<0.05 vs. S; *p < 0.05 vs. I/R; #p < 0.05 vs. D+I/R; &p < 0.05 vs. M+I/R. S: sham operation; I/R: myocardial ischemia-reperfusion; D: dexmedetomidine; M: methyllycaconitine.

Since we found that DEX activates the vagal nerve, we next investigated if the α7nAchR, which is a pivotal member in the cholinergic anti-inflammatory pathway, is involved in the vagal response. We repeated the I/R protocol with or without pretreatment with the selective α7nAChR antagonist MLA. MLA partially reversed both the short-term (M+D+I/R *vs*. D+I/R, all p < 0.05; Fig 5) and the long-term (M+D+I/R *vs*. D+I/R, p < 0.05; Fig 6) cardioprotective effects of DEX. Pretreatment with both DEX and MLA prior to I/R elevated HMGB1 mRNA levels and protein expression compared to DEX pretreatment alone (M+D+I/R *vs*. D+I/R, p < 0.05; Fig 7). Pretreatment with MLA without DEX before I/R exacerbated cardiac damage and inflammatory injury (M+I/R *vs*. I/R, p < 0.05; Figs 5 and 6). We also observed a notable increase in cardiac HMGB1 release in the M+ I/R group (M+I/R *vs*. I/R, p < 0.05, Fig 7).

### Cardiac-selective overexpression of HMGB1 impaired DEX-induced cardioprotection

**Fig 8:**
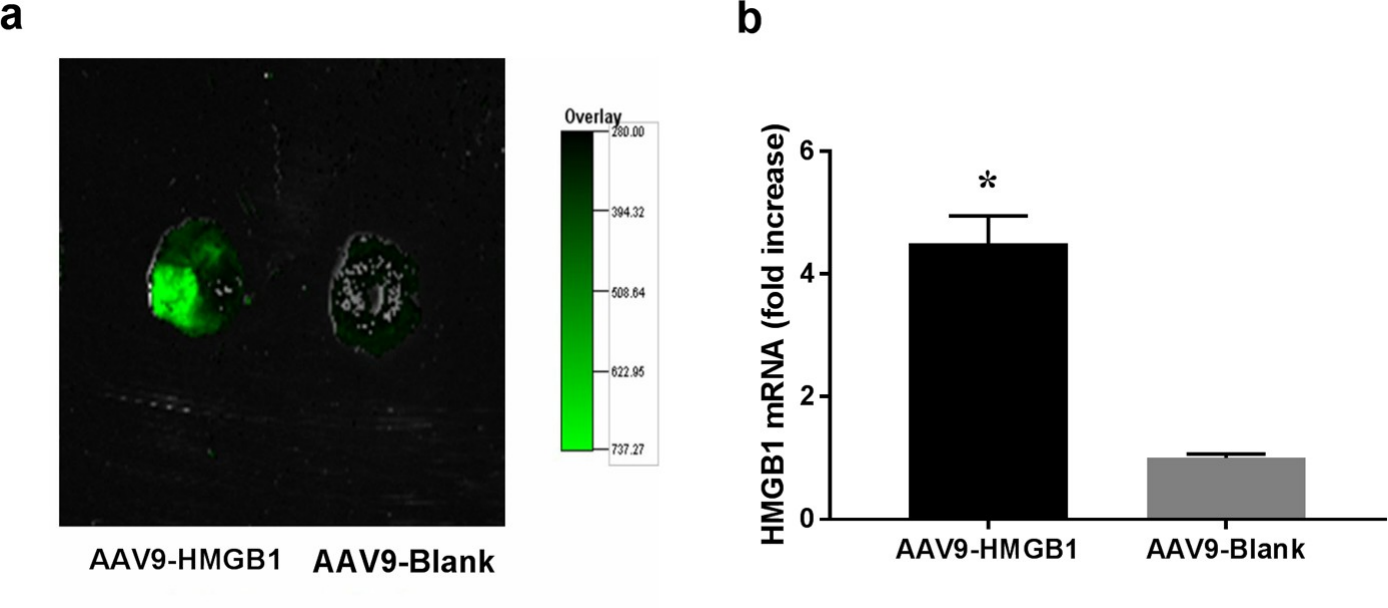
Cardiac-selective overexpression of HMGB1 using AAV9. Intrapericardial injection of AAV9-HMGB1 (conjugated to green fluorescent protein) was performed to induce cardiac-selective HMGB1 overexpression; blank AAV9 (AAV9-Blank) was used as a control. Representative photographs of heart sections from both AAV9-HMGB1 and AAV9-Blank groups are shown in panel (a); mRNA levels of myocardial HMGB1 were quantified using qPCR (b). *p < 0.01 vs. AAV9-Blank.

**Fig 9:**
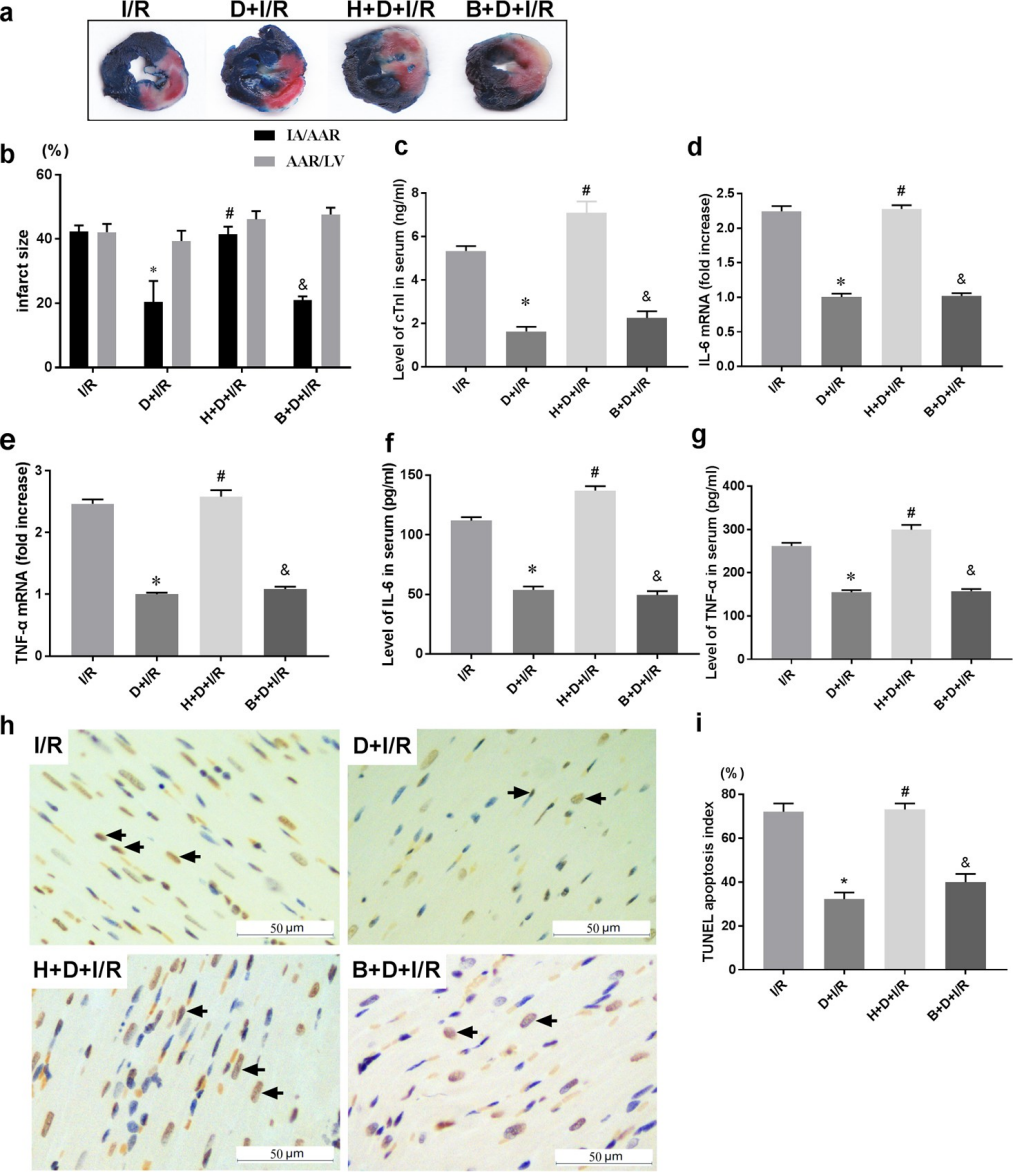
Cardiac-selective HMGB1 overexpression eliminates DEX-induced short-term cardioprotection against I/R. Rats were subjected to intrapericardial injection of AAV9-HMGB1 or AAV9-Blank as control 3 weeks before DEX pretreatment followed with the I/R operation. (a-b) Infarct size and representative images of each group are shown. (c) Serum cTnI levels; mRNA levels (d-e) and serum levels (f-g) of inflammatory cytokines including IL-6 and TNF-α were also detected. Myocardial apoptosis in infarct zone was evaluated by TUNEL staining (h-i) (arrows indicate positive cells, Scale bar = 50μm.); All specimen were obtained at the timepoint of myocardial I/R for 2hrs. Data shown are mean ± SEM; n = 10 per group; *p < 0.01 vs. I/R; #p < 0.05 vs. D+I/R; &p<0.05 vs. H+D+I/R; H: AAV9-HMGB1; B: AAV9-Blank; D: dexmedetomidine; I/R: myocardial ischemia-reperfusion;

**Fig 10:**
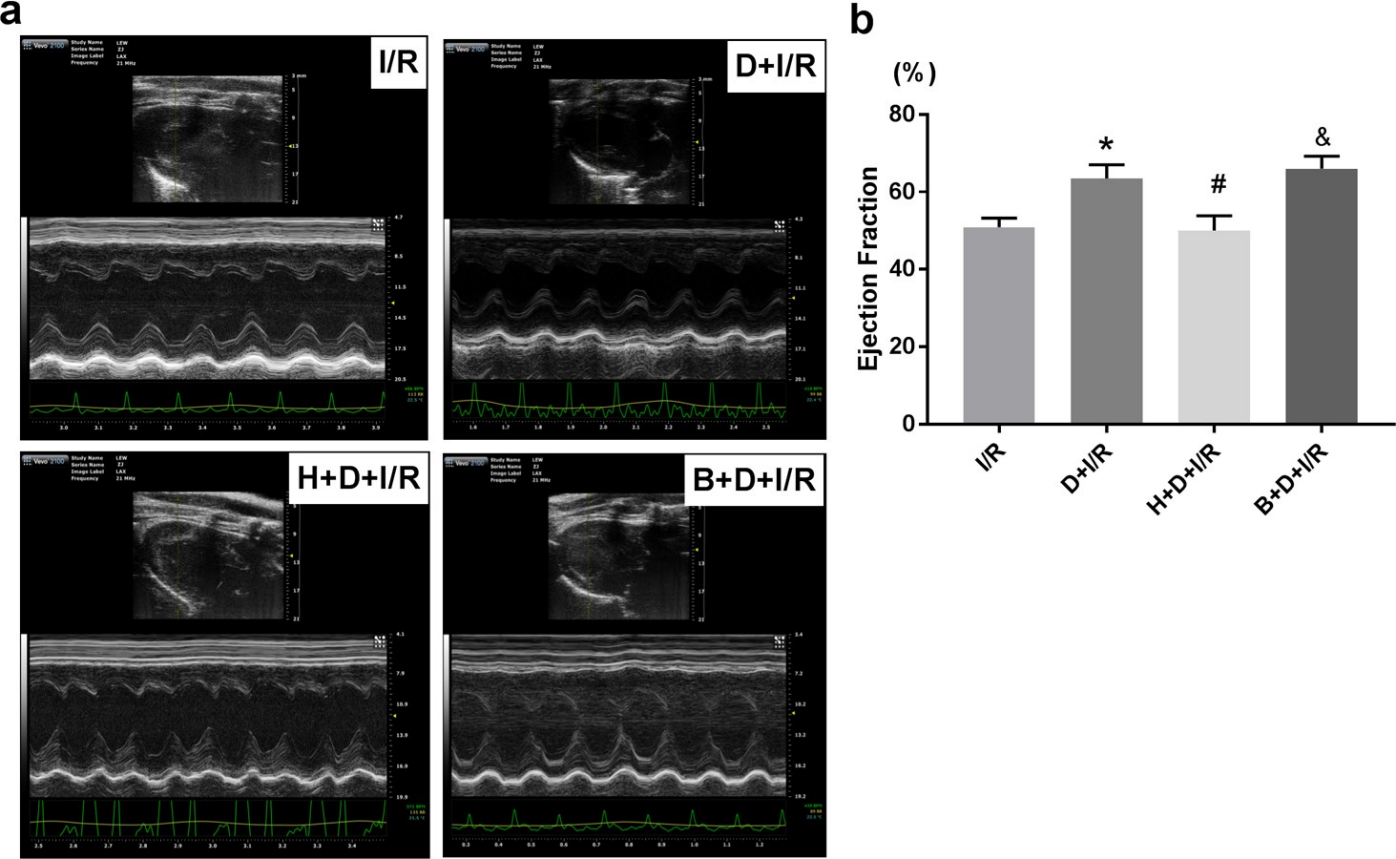
Cardiac-selective HMGB1 overexpression eliminates DEX-induced long-term cardioprotection against I/R. Rats were subjected to intrapericardial injection of AAV9-HMGB1 or AAV9-Blank as control before DEX pretreatment followed with the I/R operation. After another 4 weeks of reperfusion, the echocardiography was performed to evaluate the LV function before the animals were sacrificed. M-mode images at long-axis view were shown. Ejection fraction (EF) were calculated and compared among different groups: EF (%) = [(EDV−ESV)/EDV]×100%; EDV (end diastolic volume); ESV (end systolic volume). All measurements were based on the average of three consecutive cardiac cycles. Data shown are mean ± SEM; n = 6 per group; *p < 0.01 vs. I/R; #p < 0.05 vs. D+I/R; &p<0.05 vs. H+D+I/R; H: AAV9-HMGB1; B: AAV9-Blank; D: dexmedetomidine; I/R: myocardial ischemia-reperfusion;

The results above suggest that DEX-induced cardioprotection against I/R may be mediated by α7nAchR downregulation of HMGB1 expression in the heart. Since HMGB1 acts as an important bridging protein connecting the upstream efferent cholinergic pathway with the downstream pro-inflammatory cascade, and given that we previously demonstrated that DEX attenuates myocardial I/R injury by inhibiting the HMGB1-TLR4-MyD88-NF-κB signaling pathway [10], we hypothesized that HMGB1 is a key regulating target of DEX. To further investigate the role of HMGB1, we subjected animals to intrapericardial injection with either AAV9-HMGB1 to induce HMGB1 overexpression or AAV9-Blank as control. Following injection, cardiac HMGB1 mRNA levels were approximately 4.5-fold increase compared to the Blank control (AAV9-HMGB1 *vs*. AAV9-Blank, p < 0.05; representative images of HMGB1 cardiac expression are shown in Fig 8) DEX pretreatment failed to confer both the short-term (Fig 9) and long-term (Fig 10) cardioprotection in the HMGB1 overexpressed group (H+D+I/R *vs*. B+D+I/R, all p< 0.05). These results suggest that cardiac HMGB1 overexpression aggravates I/R injuries and eliminates DEX-induced cardioprotection, which supports our hypothesis that HMGB1 is the key target through which DEX exerts its anti-inflammatory and cardioprotecitive effects in the myocardial I/R model.

## Discussion

Using a rat myocardial I/R model, our study provides new evidences that DEX prevents cardiac damage and inflammation by inhibiting HMGB1 release from the ischemic heart. These beneficial effects require vagal nerve integrity and depend on α7nAChR-mediated cholinergic stimulation. Cardiac-selective overexpression of HMGB1 using AAV9-mediated delivery system further confirmed that HMGB1 is a crucial target through which DEX mediates its anti-inflammatory properties.

Similar to infection, sterile injuries resulting from some pathologies, including myocardial infarction (MI), hypertension and diabetes, can activate the immune system and increase cytokine and chemokine production [21–23], causing severe systemic and local inflammation that aggravates tissue damage [24]. Consistent with our previous research [9, 10], the present study demonstrated that DEX pretreatment significantly attenuated I/R-induced cardiac damage, as evidenced by decreases in short-term injury indicators including myocardial infarct size, cTnI release, myocardial apoptosis, cardiac HMGB1 expression, IL-6 and TNF-α production, as well as the improvement in long-term cardiac function at 4 weeks post-reperfusion. These findings support our hypothesis that DEX pretreatment can prevent both systematic pro-inflammatory mediator release and local inflammatory cell infiltration into cardiac tissue which contributes to heart dysfunction in a long term.

As a selective α2-adrenic receptor agonist, DEX has sedative, anxiolytic, and analgesic properties. However, the mechanism by which DEX exerts its anti-inflammatory effects has remained undefined. Recent evidences from different animal models of cerebral ischemia [25], tibial fraction [12], and endotoxemia [26, 27] indicate that DEX-mediated anti-inflammation may be relevant to the “cholinergic anti-inflammatory pathway”. Since the cholinergic anti-inflammatory pathway includes the efferent vagal nerve, the neurotransmitter acetylcholine and the α7nAchR [28], we administrated vagotomy and MLA to block the α7nAchR in combination with DEX to investigate if these factors were also involved in the DEX-mediated anti-inflammatory action. As expected, both unilateral vagotomy and blockade of α7nAchR significantly abrogated the anti-inflammatory effects of DEX, as evidenced by reduced cardiac HMGB1, IL-6, and TNF-α production. Consistently, the DEX-induced cardioprotection against I/R was also abolished by vagotomy and MLA, in aspects of cTnI release, myocardial apoptosis, infarct size as well as the LV heart function in the long-term. These results indicate that the anti-inflammatory and cardioprotective effects of DEX depend on activation of the cholinergic pathway.

At the early stage of myocardial ischemia, intracellular HMGB1, as well as other proteins, such as heat shock proteins and S100s (which are called damage associated molecular patterns, DAMPs), are passively released from necrotic cardiomyocytes[13]. DAMPs act as alarmins, notifying the immune system that there is tissue damage and initiating an early inflammatory response, which ultimately exacerbates myocardial injury [29]. Thus, inhibiting the early damage signals could be an effective approach to prevent subsequent lesions. Our previous study [5] showed that stimulating the vagal nerve by electroacupuncture *in vivo* or pretreating neonatal cardiomyocytes with acetylcholine *in vitro* inhibited ischemia or hypoxia-induced cardiac HMGB1 release through an α7nAchR-dependent mechanism. Similarly, in the present study, we observed a significant decrease in myocardial HMGB1 expression in DEX pretreated animals compared to shams. Furthermore, cardiac-selective overexpression of HMGB1 using AAV9 transfection completely abolished the anti-inflammatory effects of DEX. Together, these findings suggest that HMGB1 is a crucial target in the vagal nerve-modulated cholinergic anti-inflammatory pathway through which DEX exerts its beneficial effects.

There are some limitations in this study. First, although the present study demonstrated that DEX pretreatment inhibited early inflammation and improved cardiac dysfunction after I/R, the inflammation in myocardial I/R has been shown to be not only deleterious but also beneficial in the process of myocardial damage and remodeling. In particular, TNF-α, as a key inflammatory mediator, is reported to have a paradoxical effect including both protecting and detrimental roles in ischemia-induced heart dysfunction [30, 31]. Second, cardiac fibroblasts which constitutes the majority of non-myocyte cells in the heart, has been reported to prevent cardiac myocytes from hypothermic lesions by producing some grow factors and cytokines and interacting with other cell types [32]. Conversely, Kawaguchi et al. indicated that inflammasome activation in myofibroblast contributes to the I/R injury [18]. Thus, the role of fibroblast in myocardial I/R remains controversy and uncertain. In our present study, we cannot demonstrate whether myofibroblast takes part in the protective effects of DEX. Third, the precise mechanism by which DEX either directly or indirectly interacts with the cholinergic anti-inflammatory pathway are still unknown. Sharp et al. reported that dexmedetomidine decreases both GABAergic and glycinergic inhibitory input to cardiac vagal neurons, which result in decreased central sympathetic output as well as increased parasympathetic output from brainstem cardiac vagal neurons[33]. Further experiments are required to verify this possibility. Lastly, we overexpressed cardiac HMGB1 using AAV9 transfection to investigate if HMGB1 is the target protein through which DEX exerts its cardioprotection. An HMGB1-knockout animal model could better elucidate this mechanism; however, it has been reported that HMGB1 knockout is embryonically lethal in mice[34] indicating that HMGB1 is vital. Therefore, future studies could develop a cardiac conditional HMGB1 knockout mouse model to further investigate the effects of DEX-induced cardioprotection.

## Conclusions

The present suggests that DEX pretreatment protects the heart against I/R injury by inhibiting cardiac HMGB1 production via activating the cholinergic anti-inflammatory pathway. This study identified the upstream mechanism of the HMGB1-TLR4-MyD88-NF-κB pathway, which was previously used to investigate DEX-induced cardioprotection, offering us a better understanding of DEX’s anti-inflammatory effects.

## Supporting information

S1 Fig Map of the plasmid vector applied for cardiac-selective HMGB1 overexpression. The HMGB1 cDNA sequence conjugated to green fluorescent protein (GFP) was cloned into a pAAV ITR-containing plasmid driven by the CMV promoter. See more details for the vector: https://www.abmgood.com/vectors/vectorDisplay.php?vec=pAAV-G-CMV-2A-GFP&page=seq
(TIF)

S2 Fig No significant differences of myocardial fibrosis were observed at the timepoint of 2hrs post-I/R. Prius red staining was performed to assess the myocardial fibrosis among all experimental groups. However, it seemed the fibrosis is not that obvious at our observing time point (ischemia for 30min and reperfusion for 2 hrs) and there were no significant differences among all groups. Scale bar = 50μm. n = 3 per group; S: sham operation; I/R: myocardial ischemia-reperfusion; D: dexmedetomidine; V: vagotomy; M: methyllycaconitine; H: AAV9-HMGB1; B: AAV9-Blank.
(TIF)

S3 Fig Vagotomy or Dex pretreatment in basic condition has no influences on myocardial HMGB1 and cytokines expression. The animals were subjected to either unilaterally vagotomy or Dex-pretreatment (same dose as before). After 2.5 hrs, they were sacrificed and the myocardial levels of cytokines (IL-6 and TNF-α) and HMGB1 were detected by qPCR and western blot (All p>0.05). Ctrl: normal animals; Dex: dexmedetomidine; VG:vagotomy;
(TIF)

S4 Fig Survival analysis of animals 28 days post-I/R among all experimental groups. 90 animals were used for survival estimation (n = 10 per group). Survival probabilities were calculated with the use of Kaplan-Meier methods and compared with the use of a log-rank test (Log-Rank test, p = 0.775). S: sham operation; I/R: myocardial ischemia-reperfusion; D: dexmedetomidine; V: vagotomy; M: methyllycaconitine; H: AAV9-HMGB1; B: AAV9-Blank.
(TIF)
