# Supplementary figures and images for: Dexmedetomidine-induced cardioprotection is mediated by inhibition of high mobility group box-1 and the cholinergic anti-inflammatory pathway in myocardial ischemia-reperfusion injury
Source: PLoS One. 2019 Jul 25;14(7):e0218726. doi: 10.1371/journal.pone.0218726 (PMC6657822; doi:10.1371/journal.pone.0218726)

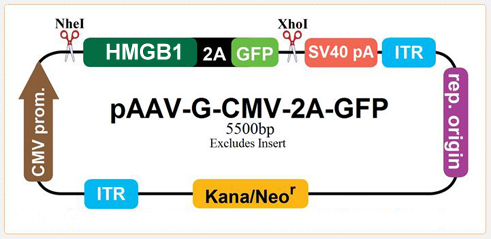

Supplement: S1 Fig — The HMGB1 cDNA sequence conjugated to green fluorescent protein (GFP) was cloned into a pAAV ITR-containing plasmid driven by the CMV promoter. See more details for the vector: https://www.abmgood.com/vectors/vectorDisplay.php?vec=pAAV-G-CMV-2A-GFP&page=seq (TIF) [file pone.0218726.s001.tif]

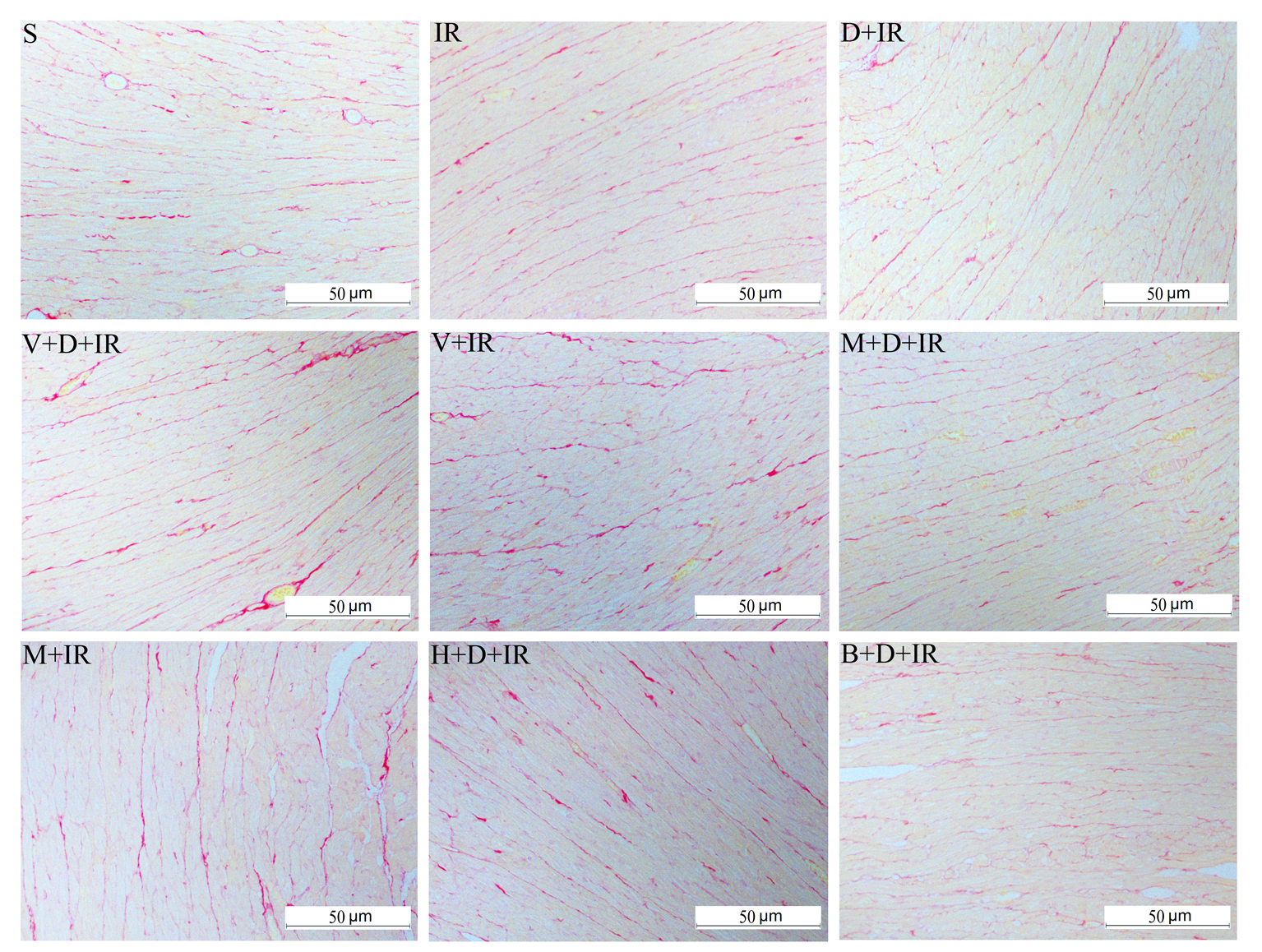

Supplement: S2 Fig — Prius red staining was performed to assess the myocardial fibrosis among all experimental groups. However, it seemed the fibrosis is not that obvious at our observing time point (ischemia for 30min and reperfusion for 2 hrs) and there were no significant differences among all groups. Scale bar = 50μm. n = 3 per group; S: sham operation; I/R: myocardial ischemia-reperfusion; D: dexmedetomidine; V: vagotomy; M: methyllycaconitine; H: AAV9-HMGB1; B: AAV9-Blank. (TIF) [file pone.0218726.s002.tif]

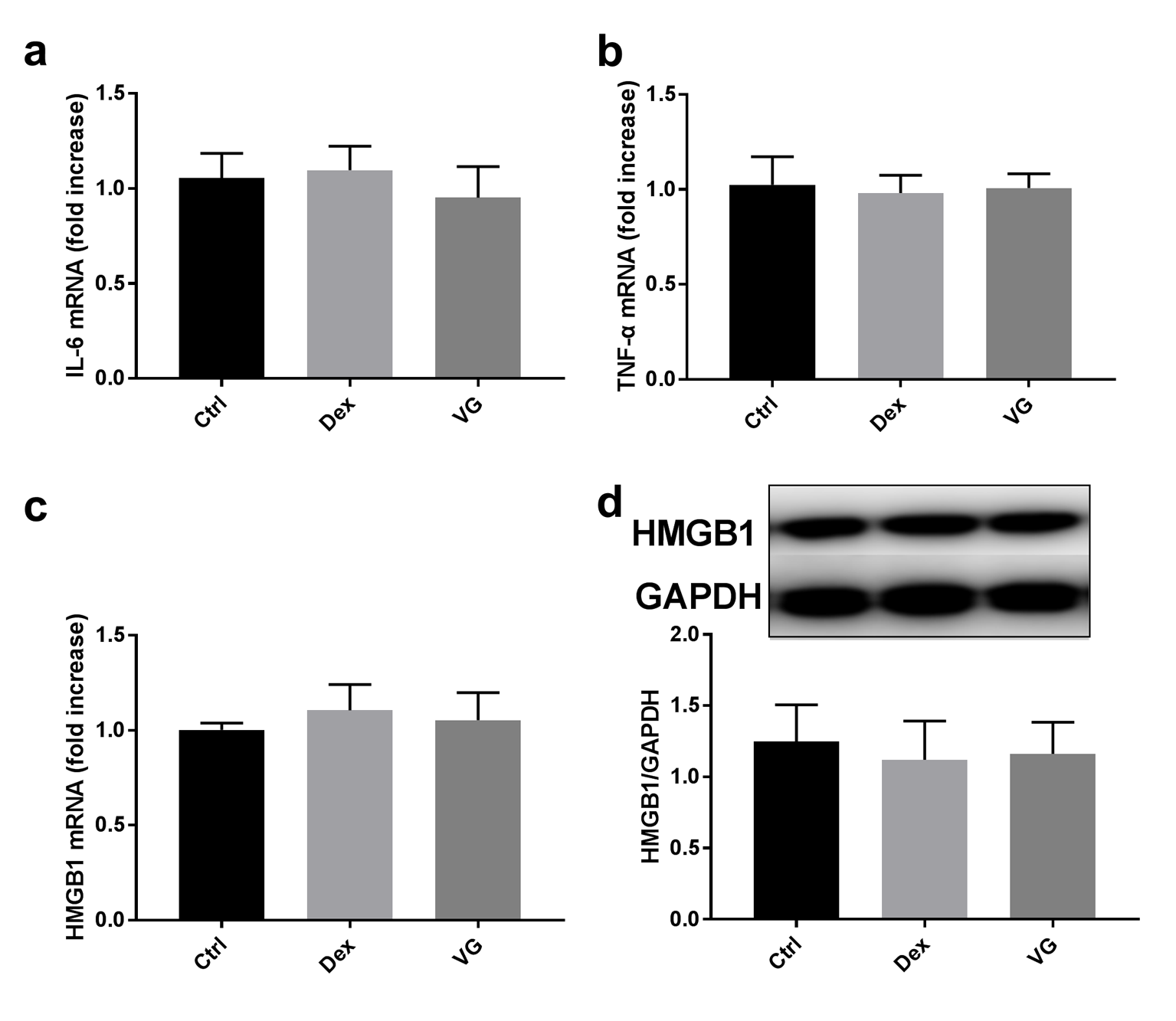

Supplement: S3 Fig — The animals were subjected to either unilaterally vagotomy or Dex-pretreatment (same dose as before). After 2.5 hrs, they were sacrificed and the myocardial levels of cytokines (IL-6 and TNF-α) and HMGB1 were detected by qPCR and western blot (All p>0.05). Ctrl: normal animals; Dex: dexmedetomidine; VG:vagotomy; (TIF) [file pone.0218726.s003.tif]

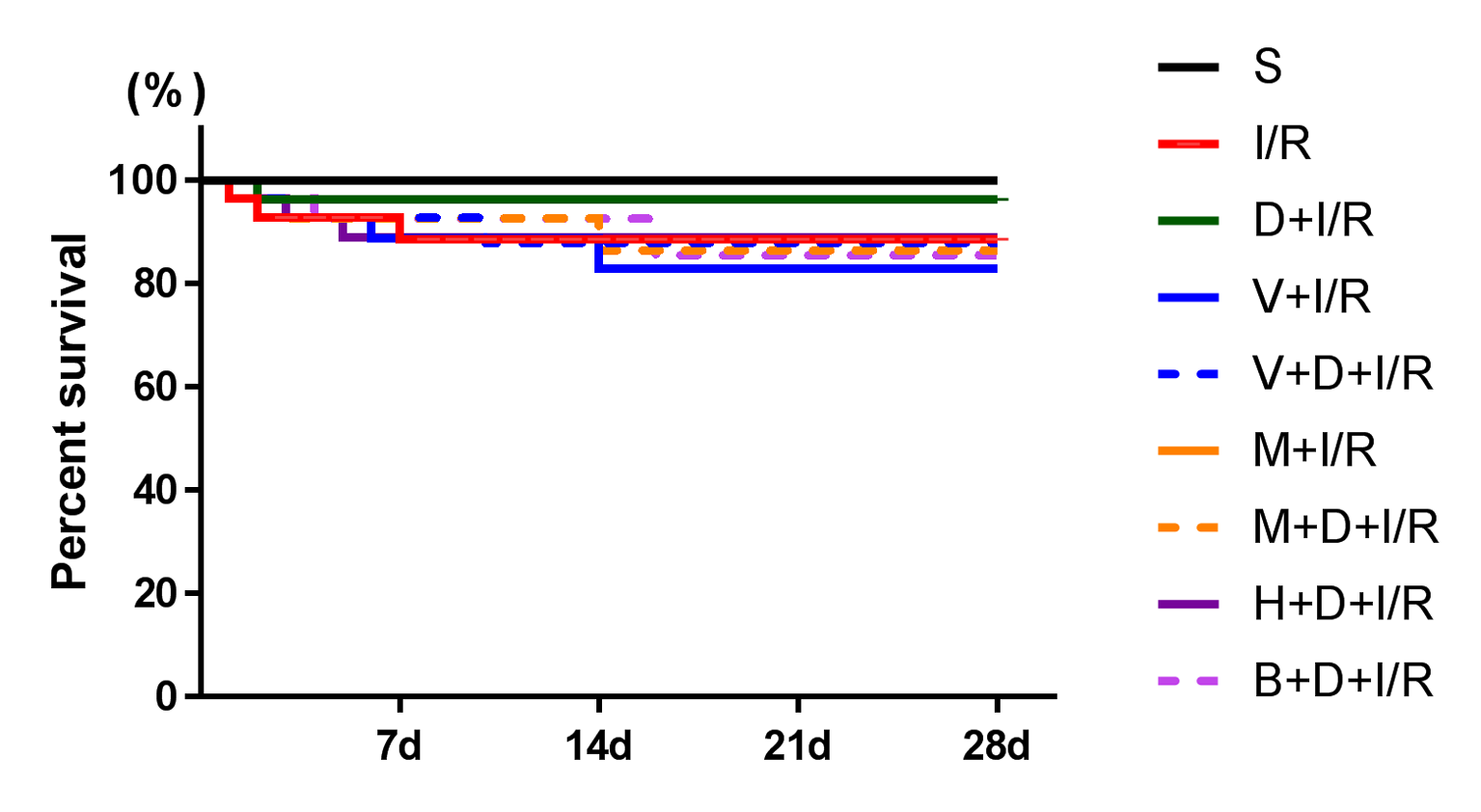

Supplement: S4 Fig — 90 animals were used for survival estimation (n = 10 per group). Survival probabilities were calculated with the use of Kaplan-Meier methods and compared with the use of a log-rank test (Log-Rank test, p = 0.775). S: sham operation; I/R: myocardial ischemia-reperfusion; D: dexmedetomidine; V: vagotomy; M: methyllycaconitine; H: AAV9-HMGB1; B: AAV9-Blank. (TIF) [file pone.0218726.s004.tif]
